# The Identification and Management of High Blood Pressure Using Exercise Blood Pressure: Current Evidence and Practical Guidance

**DOI:** 10.3390/ijerph19052819

**Published:** 2022-02-28

**Authors:** Martin G. Schultz, Katharine D. Currie, Kristofer Hedman, Rachel E. Climie, Andrew Maiorana, Jeff S. Coombes, James E. Sharman

**Affiliations:** 1 Menzies Institute for Medical Research, University of Tasmania, Hobart, TAS 7000, Australia; rachel.climie@utas.edu.au (R.E.C.); james.sharman@utas.edu.au (J.E.S.); 2 Department of Kinesiology, Michigan State University, East Lansing, MI 48824, USA; curriek4@msu.edu; 3 Department of Clinical Physiology in Linköping, and Department of Health, Medicine and Caring Sciences, Linköping University, 58183 Linköping, Sweden; kristofer.hedman@liu.se; 4 Faculty of Health Sciences, School of Physiotherapy and Exercise Science, Curtin University and Allied Health Department, Fiona Stanley Hospital, Perth, WA 6102, Australia; a.maiorana@curtin.edu.au; 5 School of Human Movement and Nutrition Sciences, University of Queensland, Brisbane, QLD 4072, Australia; jcoombes@uq.edu.au

**Keywords:** exercise physiology, exercise testing, blood pressure, cardiovascular disease

## Abstract

High blood pressure (BP) is a leading risk factor for cardiovascular disease (CVD). The identification of high BP is conventionally based on in-clinic (resting) BP measures, performed within primary health care settings. However, many cases of high BP go unrecognised or remain inadequately controlled. Thus, there is a need for complementary settings and methods for BP assessment to identify and control high BP more effectively. Exaggerated exercise BP is associated with increased CVD risk and may be a medium to improve identification and control of high BP because it is suggestive of high BP gone undetected on the basis of standard in-clinic BP measures at rest. This paper provides the evidence to support a pathway to aid identification and control of high BP in clinical exercise settings via the measurement of exercise BP. It is recommended that exercise professionals conducting exercise testing should measure BP at a fixed submaximal exercise workload at moderate intensity (e.g., ~70% age-predicted heart rate maximum, stage 1–2 of a standard Bruce treadmill protocol). If exercise systolic BP is raised (≥170 mmHg), uncontrolled high BP should be assumed and should trigger correspondence with a primary care physician to encourage follow-up care to ascertain true BP control (i.e., home, or ambulatory BP) alongside a hypertension-guided exercise and lifestyle intervention to lower CVD risk related to high BP.

## 1. Introduction

High blood pressure (BP) is the leading risk factor for cardiovascular disease (CVD) [1]. Worldwide, more than one billion adults have high BP [2] with many more either unaware they have the condition or inadequately controlled with medication [3]. Adding to this, conventional methods of BP assessment conducted in a clinical (office-based) setting at rest fail to identify all cases of high BP, with 8–20% of individuals with normal clinic BP having raised out-of-clinic BP (i.e., masked uncontrolled hypertension). This highlights that many people will have suboptimal BP management, placing them at increased CVD risk [4]. Thus, there is a need for new settings for the identification and management of high BP.

There is growing evidence for the clinical and prognostic utility of abnormally high BP responses to exercise [5,6,7]. Research suggests that an exaggerated exercise BP (EEBP) may signal high BP that has gone undetected with resting measures in the clinic by revealing the presence of underlying or masked hypertension [8,9,10]. However, EEBP is not generally considered a prognostic indicator because exercise BP is not routinely measured in primary care settings.

Clinical exercise professionals (e.g., exercise physiologists and physical therapists) are usually considered as allied health professionals who specialise in exercise testing and/or prescription for chronic disease. In Australia alone, >300,000 referrals are made to an exercise physiologist each year [11]. Patient consults will usually involve an exercise test to assess functional capacity, where BP can be measured. Thus, clinical exercise professionals are well positioned to (1) identify individuals at CVD risk from abnormally high BP responses to exercise that would otherwise be missed by conventional (i.e., resting) screening methods (in Australia alone, this would be up to 60,000 individuals each year and many millions worldwide); (2) provide correspondence and referral to primary care physicians for definitive assessment of BP control using out-of-office BP methods; and (3) begin lifestyle interventions, including exercise, as a frontline treatment to reduce the risk related to high BP as well as improve other comorbidities.

This paper presents the current evidence surrounding the clinical value of EEBP, supporting best-practice measurement of exercise BP in clinical exercise settings. The primary aim is to provide practical guidance on the correct measurement and interpretation and pathways to promote the identification and management of high BP via exercise BP measurement in this setting.

### 1.1. The Blood Pressure Response to Acute Dynamic Incremental Exercise

Dynamic incremental exercise causes increased demand for blood delivery to support the metabolic processes within active muscular regions. This demand is normally met by elevation in cardiac output (the principal driver of aerobic capacity) [12] and reductions in systemic vascular resistance during large muscle group activity [13]. The reduction in systemic vascular resistance sustains diastolic BP at a relatively constant level during exercise; however, the rising cardiac output drives elevations in systolic BP proportional to exercise intensity and workload. Systolic BP will normally continue to rise as exercise intensity increases in most (but not all) people, plateauing before maximal aerobic capacity is reached. The rate of systolic BP increase will vary based on age, sex, ethnicity, cardiorespiratory fitness, and overall health status [14]. Consequently, peak or maximum systolic BP will in part be related to the peak or maximal workload at which the BP measurement was made. Current guidelines from the American College of Sports Medicine (ACSM) outline a systolic BP value of 250 mmHg as a relative indication to terminate clinical exercise testing on the balance of safety [15]. However, this BP threshold is arbitrary, does not account for exercise workload, and lacks justification via empirical evidence.

Irrespective of BP level and control at rest, some people may have an EEBP response to clinical exercise testing. Thresholds to denote EEBP have traditionally been set based on exceeding 90th centile BP responses to maximal or peak intensity exercise, and commonly exercise BP values ≥ 210/100 mmHg for males and ≥190/100 mmHg for females [16]. This is because some studies have shown relationships with CVD outcomes with values beyond these levels [17,18]. However, EEBP of pathological significance can occur at any exercise intensity [5,19], with research suggesting that EEBP at submaximal exercise intensities may hold stronger associations with CVD risk [5,20].

### 1.2. Evidence Supporting the Clinical Value of Submaximal Exercise BP Measurement

It is beyond the scope of this practical guiding document to extensively review the literature surrounding exercise BP and CVD risk, but readers are referred to previous scientific reviews on the topic [21,22,23,24]. Briefly however, Schultz et al. conducted the first meta-analysis that described the relationship between EEBP during submaximal exercise (of various workloads and intensities) and CVD risk in those with normal resting BP [5]. The pooled analysis indicated EEBP during various intensities of submaximal exercise was associated with a 36% increased cardiovascular event and mortality rate, with each 10-mmHg increase in systolic BP during submaximal exercise intensities associated with a 4% increased event rate independent of resting BP, age, sex, and other CVD risk factors [5]. More recent longitudinal studies and a further meta-analysis have confirmed the CVD risk associated with EEBP during submaximal exercise [25,26,27].

BP recorded during submaximal exercise of a moderate intensity is likely indicative of the true burden of high BP because it is an intensity of exercise commensurate with some daily life “ambulatory” activities (e.g., climbing stairs, rushing to catch a bus). Indeed, EEBP during moderate intensity exercise is associated with the development of hypertension in populations without high resting BP [20,26]. Moreover, submaximal exercise BP recorded during moderate intensity exercise has been shown to expose BP-related risk undetected by standard in-clinic measurement of BP at rest [10], revealing [8,9,28] or ruling out [29] the presence of masked hypertension. This is an important clinical finding since masked hypertension carries similar CVD risk to established hypertension [30] and may occur in up to 20% of the general population [4], with greater prevalence in those with established disease, such as type 2 diabetes (>30%) [31]. It is also known that EEBP appears to cluster with other high-BP related CVD risk factors [32]. Several studies have also shown relationships between EEBP and adverse cardiac structure [33,34,35,36] and function [37], indicative of hypertensive heart disease. Sarma et al. also recently reported EEBP during submaximal exercise to be related to increased left ventricular and aortic stiffness in otherwise healthy middle-aged women, proposing that EEBP could serve as a sentinel for heart failure with preserved ejection fraction [38]. An additional benefit of measuring BP at a fixed submaximal workload rather than peak/maximal BP is that this circumvents the confounding effect of workload and cardiorespiratory fitness [21]. Although there are some evidence gaps, the above data strongly suggest that EEBP recorded at moderate intensity likely provides clinically useful information about high BP and related CVD risk. Thus, careful and standardized measurement of exercise BP by clinical exercise professionals has significant potential to influence the ongoing care of people with uncontrolled high BP.

### 1.3. A Pathway for the Identification and Management of High BP via Exercise BP Measurement within Clinical Exercise Settings

**Figure 1:**
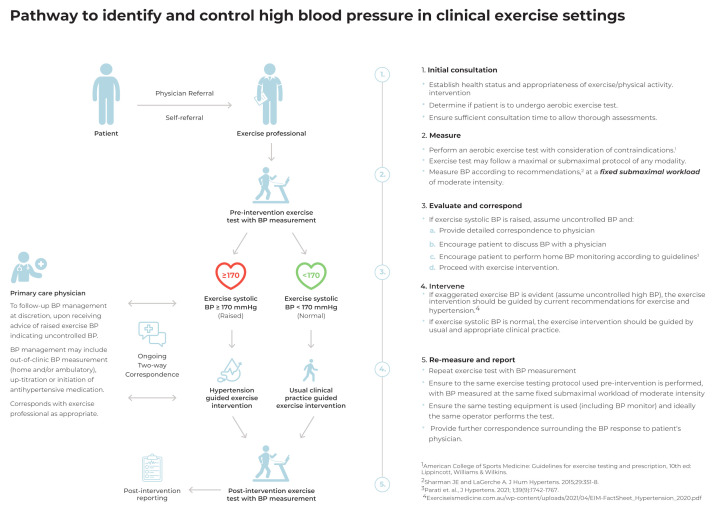
Pathway to identify and control high blood pressure in clinical exercise settings.

The following section outlines a pathway, recommendations, and associated evidence for the identification and control of high BP within clinical exercise settings, with key points also summarised as an infographic (Figure 1). The infographic is also included as a Supplementary Materials for practical use (i.e., printing and display) for exercise professionals.

## 2. Initial Consultation

### Key Recommendations

- Establish health status and appropriateness for exercise/physical activity intervention;
- Determine if patient is to undergo an aerobic exercise test;
- Ensure sufficient consultation time to allow thorough assessments.

An initial consultation with a clinical exercise professional will typically begin via either a direct referral from a primary care physician, or simple “walk-in” self-referral. Primary care physician referral systems differ substantially worldwide with country-specific health system structures and associated patient costs/reimbursement for clinical exercise services. However, referral to a clinical exercise professional for exercise (and other lifestyle) intervention is usually indicated (and made) due to the presence of a chronic health condition or injury. Whilst individual patient history and presence of a chronic condition or injury should dictate the clinical approach to be taken by the exercise professional, it is important to note that a clinical pathway to identify and manage high BP is suitable to follow irrespective of whether the patient referral is specific to the management of high BP (hypertension). Indeed, whether the patient has established hypertension or not, the measurement of exercise BP is an opportunistic point in health care delivery to screen for high BP that may not otherwise occur.

Medical clearance to undertake exercise training may be assumed with a direct physician referral; however, exercise professionals with advanced training in exercise prescription are qualified to determine the suitability and participant readiness for an exercise intervention with/without the need for additional medical clearance [39]. Several endorsed “tools” are available to aid in the decision process (e.g., ACSM preparticipation screening algorithm [40], the Exercise and Sports Science Australia (ESSA) Pre-Exercise Screening System (https://www.essa.org.au/Public/ABOUT_ESSA/Pre-Exercise_Screening_Systems.aspx), and the Canadian Physical Activity Readiness Questionnaire for Everyone (PAR-Q+)) [41]. Nonetheless, clinical judgment should be used and correspondence with a qualified medical clinician remains appropriate in most circumstances.

It may not always be practical to undertake an objective measurement of aerobic fitness via an exercise test at an initial consultation due to contraindications to exercise testing. However, the benefits of obtaining reliable and objective baseline information surrounding aerobic fitness combined with the additional opportunity to screen for BP abnormalities (as proposed in this review) should outweigh any self-imposed time constraints.

## 3. Measure

### Key Recommendations

- Perform an aerobic exercise test with consideration of contraindications;
- Exercise test may follow a maximal or submaximal protocol of any modality;
- Measure BP according to recommendations at a fixed submaximal workload of moderate intensity.

It is beyond the scope of this review to provide extensive detail surrounding clinical exercise testing procedures and protocols. This information is outlined within the ACSM and American Heart Association (AHA) guidelines for exercise testing [15,42]. However, the choice of test and protocol should consider the objectives of the test and the information required for exercise programming whilst taking into consideration any contraindications to testing. For the detection of uncontrolled high BP, the exercise modality of the test is not important since EEBP recorded from all modes of exercise testing (e.g., during a graded treadmill or cycle test or immediately following a six-minute walk test or step test) has shown association with CVD outcomes [5,20]. While maximal exercise testing is useful in some populations to obtain an objective measure of peak physiological and exercise performance, it is not required for the assessment of risk related to high BP.

Clinically, the optimal intensity to assess CVD risk related to EEBP will be at a submaximal, moderate intensity of exercise. A moderate intensity of exercise will differ between individuals of various fitness levels and health status but can generally be considered (for example) stages 1–2 of a standard Bruce treadmill protocol or approximately 70% of age-predicted maximal heart rate [43]. Submaximal exercise tests conducted up to or at a moderate exercise intensity may provide greater accessibility to patients with chronic disease or injury can be performed at a lower cost and may (depending on the patient population studied) require less technical/professional supervision compared with maximal exercise testing [44]. Importantly, measurement of BP during moderate-intensity exercise is also comparatively easy because it removes the influence of external artefacts (noise and movement) that can occur during higher intensity exercise [24]. Some studies have also found substantial test-retest reliability of EEBP classification during early exercise testing stages [45], further enhancing clinical applicability.

A crucial consideration towards optimal measurement of exercise BP is that the workload should be “fixed” at a moderate intensity. Workloads cannot be fixed at maximal or peak exercise intensity, but it is possible during submaximal exercise of moderate intensity [21]. While it is acknowledged that the relative intensity will differ between individuals for a given fixed workload, there is a strong association between workload (e.g., watts) and systolic BP [46]. Thus, the correct clinical interpretation of EEBP will be influenced by the workload at the time of BP measurement [21]. Fixing the workload rather than intensity will remove the influence of inter-individual differences in aerobic fitness on the BP response. Someone with a higher relative aerobic capacity should, for a given workload, record a lower BP (a physiological response) compared with an unfit individual who may record a higher relative BP (a pathological response) [21]. There is currently no evidence available to suggest the most appropriate workload to elicit EEBP and reveal uncontrolled BP. Therefore, the exercise professional should use clinical judgment in selecting an appropriate workload that elicits a moderate intensity. It may also be appropriate to choose a different fixed workload for BP measurement according to characteristics of the patients being studied (e.g., 75 watts for those aged over 60 or 100 watts for those with above average aerobic fitness). The key is to ensure it is consistently applied within a single clinic to allow appropriate clinical interpretation of EEBP.

**Figure 2:**
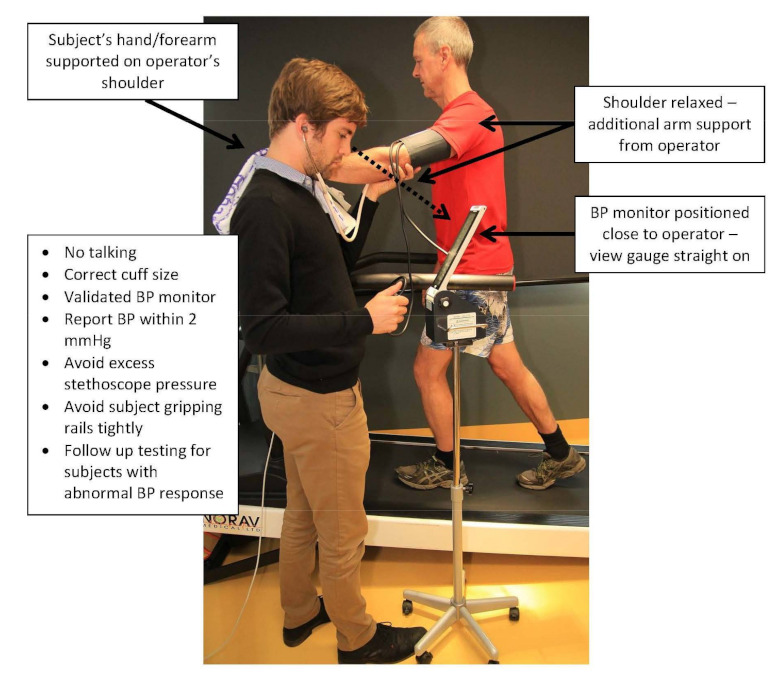
Appropriate blood pressure (BP) measurement by manual auscultation during exercise stress testing from Sharman et al. [24], reproduced with permission from Springer Nature.

Guidelines recommend that BP measurement should take place before the exercise test, at every stage of exercise (including peak/maximal intensity if such a test is performed), and in recovery from an exercise test [15,42]. Manual auscultation remains the recommended approach [15,42] although there are automated, motion-tolerant devices in widespread clinical use that have shown reasonable concordance with manual or invasive exercise BP measures [47,48]. As with best-practice measurement of BP under resting conditions, operators must follow a standardized protocol to measure exercise BP because deviation from recommended protocols will increase the likelihood of BP measurement error [49]. Clinical exercise professionals should familiarize themselves with the correct measurement protocol as outlined in Table 1 and Figure 2 [24].

## 4. Evaluate and Correspond

### Key Recommendations

- If exercise systolic BP is raised (≥170 mmHg), assume uncontrolled BP and
  - Provide detailed correspondence to physician;
  - Encourage patient to discuss BP with a physician;
  - Encourage the patient to perform home BP monitoring according to guidelines;
  - Proceed with exercise intervention.

There is currently a lack of consensus around a threshold of exercise BP that denotes increased CVD risk [14]. Some studies have shown exercise systolic BP values beyond as little as 150 mmHg during moderate intensity exercise to be associated with the presence of hypertension (confirmed by ambulatory BP monitoring) [10] and risk of left ventricular hypertrophy [35]. Mariampillai et al [7] recently described a linear association between submaximal exercise systolic BP (recorded at a workload of 100 watts) and coronary artery disease risk beyond a systolic BP value of 165 mmHg. Other studies report exercise systolic BP ranging from >160–170 mmHg recorded at various submaximal exercise workloads and relative intensities to be associated with increased CVD risk [9,50]. An exercise systolic BP threshold of ≥170 mmHg vs. <130 mmHg during treadmill exercise testing (stage 2 of a Bruce protocol) was recently shown to be associated with increased risk of major adverse cardiovascular events (adjusted hazard ratio 1.33 (1.01–1.76)) [51]. This study was conducted among 14,792 patients without known cardiovascular disease at baseline and represents the most definitive evidence of a prognostically relevant threshold of exercise systolic BP at a moderate intensity of exercise. Some studies have also explored a delta systolic BP (change in systolic BP from rest to a given exercise stage/workload) as thresholds to define EEBP associated with CVD outcomes [52,53]. However, delta systolic BP is somewhat dependent on the resting BP level and usually reported as the change in systolic BP from rest to peak exercise intensity. Thus, there is currently insufficient evidence to suggest a universal delta systolic BP cut-point value. Whilst further studies aiming to determine submaximal exercise BP thresholds are awaited, we conservatively propose a submaximal exercise systolic BP cut-point of 170 mmHg to denote EEBP and signal uncontrolled high BP. Although needing definitive confirmation, this threshold of exercise systolic BP was determined from available data as a safe “middle ground” with respect to reducing false positive and false negative cases of uncontrolled high BP. This recommendation is only at the level of expert opinion but at least provides a useful starting point to trigger the BP management pathway. Most importantly, if BP is measured at a fixed workload, this should remove the influence of relative differences in aerobic fitness and health status [21].

**Figure 3:**
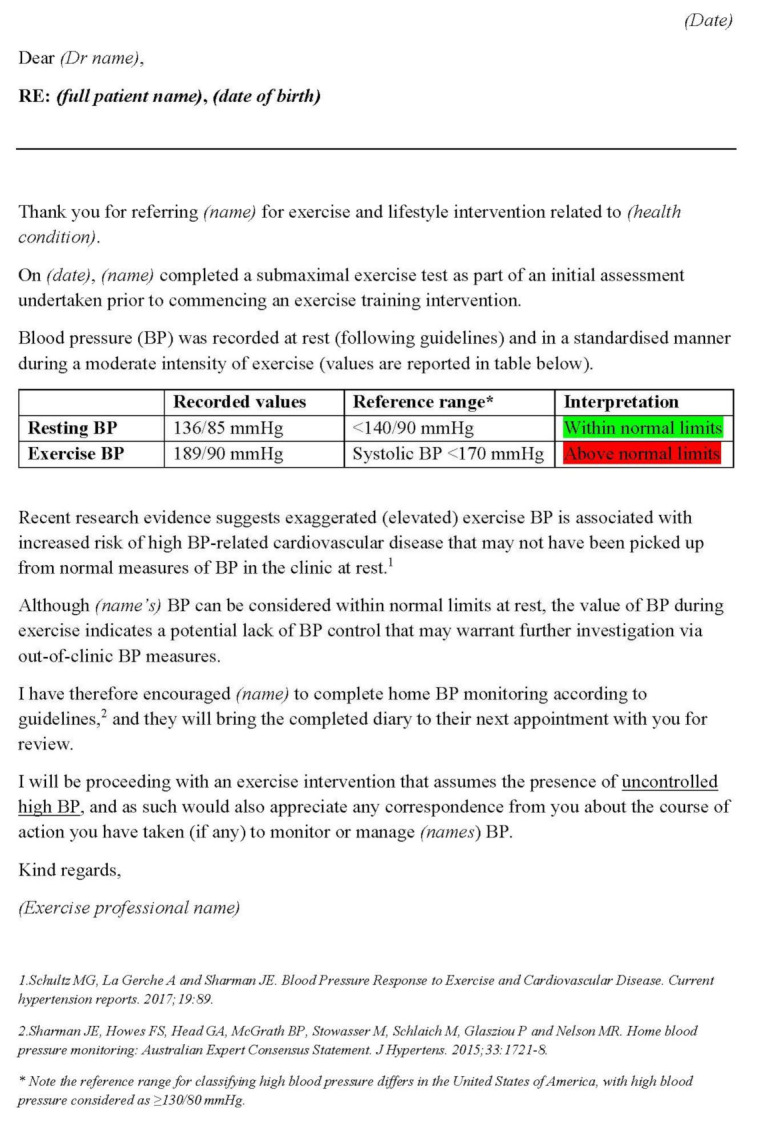
Example correspondence from an exercise professional to a primary care physician outlining findings related to exaggerated exercise blood pressure signalling uncontrolled high BP.

If an exercise systolic BP ≥ 170 mmHg at a submaximal workload of moderate intensity is measured, detailed correspondence to the patient’s referring (or regular) physician should be made. It is not the role of the exercise professional to suggest a particular course of action to the primary care physician; rather, any correspondence should be kept brief and factual, drawing attention to the exercise BP response as being raised and thus indicative of either poor BP control or a risk of future development of hypertension. This correspondence should outline (as a minimum) the tests/measurements completed, the BP values measured at rest, the BP values measured during submaximal (moderate)-intensity exercise, and information about the proposed exercise/lifestyle intervention. An example letter with proposed wording for such correspondence is provided as Figure 3.

A signal of poor BP control indicated from the exercise test result may be because of reliance on in-clinic BP measurement used to guide management. As such, the patient should be encouraged by the exercise professional to undertake home BP monitoring to confirm BP level. Home BP monitoring, when performed according to recommendations, can provide complementary information surrounding BP control to that of clinic and ambulatory BP [54,55]. User-friendly resources and instructions on how to accurately perform home BP measurement in a standardised manner are available (https://www.hbprca.com.au/wp-content/uploads/2021/08/Home-BP-monitoring-infographic.pdf and https://guidelines.hypertension.ca/wp-content/uploads/2021/05/HC-BP-Postcard.pdf) and summarised in Table 2. Tools to ensure the chosen BP measurement device for home BP monitoring is validated for accuracy are also available (https://www.menzies.utas.edu.au/documents/pdfs/Blood-pressure-devices.pdf and https://www.validatebp.org/ and https://hypertension.ca/bpdevices) [56]. Patients should be encouraged to measure BP at home for 7 days (minimum 5) at the same time each day in the morning and the evening. Each time BP is measured, two readings should be performed with one minute between each. Values should be recorded in a diary and taken to a primary care physician for interpretation.

## 5. Intervene

### Key Recommendations

- If EEBP is evident (assume uncontrolled high BP), the exercise intervention should be guided by current recommendations for exercise and hypertension;
- If exercise systolic BP is normal, the exercise intervention should be guided by usual and appropriate clinical practice.

Physical inactivity is a contributory risk factor for hypertension, with every hour spent sedentary each day associated with a 2% increased hypertension risk [57]. Alternatively, a one-MET improvement in aerobic fitness is associated with an 8% hypertension risk reduction in a dose-response relationship (i.e., greater fitness, lower hypertension risk) [58]. Thus, there is clinical impetus to promote ongoing physical activity and exercise training in all individuals (whether BP is normal or uncontrolled) to reduce the risk of developing high BP and associated CVD events. Nonetheless, a critical outcome of measuring exercise BP is that if exercise systolic BP is ≥170 mmHg, the exercise professional should assume the patient has uncontrolled high BP and provide clinical intervention accordingly.

There is substantial evidence supporting the antihypertensive effects of structured exercise training on BP. Cornelissen et al. completed a meta-analysis indicating that dynamic endurance exercise training interventions of a moderate-to-vigorous intensity may reduce systolic BP by up to 8 mmHg [59]. Of note, greater reductions were achieved in those with established hypertension (a clinic BP ≥ 140/90 mmHg). It has also been noted that specific resistance/strength training regimes also leads to meaningful reductions in systolic BP (up to 3 mmHg) [59,60]. A further meta-analysis by Cornelissen et al. indicated clinically meaningful reductions in daytime ambulatory BP can be achieved with endurance exercise training [61]. Given the intrinsic relationship between submaximal exercise BP and ambulatory BP, these results could be somewhat extrapolated to the context of EEBP. Indeed, although there is currently a paucity of data available with respect to the effect of exercise interventions on submaximal exercise BP, one randomized controlled study has shown significant reductions in systolic BP measured at stages 3 and 4 of a modified Bruce protocol compared to a non-exercise control group [62]. It is also pertinent that exercise training can provide reductions in BP that are equivalent to pharmacological intervention [63], with the BP-lowering effects of exercise training enhanced when combined with pharmacotherapy [64]. Exercise training that improves cardiorespiratory fitness should also lead to improved systolic BP for a given fixed submaximal workload and theoretically lessen the rate of increase in BP during incremental exercise testing (although this likely depends on the health status of the individual) [21].

The recommendation for exercise prescription for those with exercise systolic BP ≥170 mmHg is that the exercise professional should follow *Frequency*, *Intensity*, *Time*, *Type* principles specific to those with hypertension and as set out in the ACSM and ESSA position statements [65,66] summarised by “Exercise is Medicine” (http://exerciseismedicine.com.au/wp-content/uploads/2021/04/EIM-FactSheet_Hypertension_2020.pdf). Exercise training should involve a *frequency* of activity on most if not all days per week, with an *intensity* beginning at a light-to-moderate intensity, building to more vigorous activities once the training program is established; *time* duration of individual sessions that are 30 min or more of continuous or intermittent activity; and a *type* of exercise that is predominantly aerobic/endurance based (e.g., walking, cycling, or running), supplemented often (e.g., two days per week) with resistance based exercise since this form of exercise is also considered efficacious in the lowering of BP [60]. High-intensity interval training (HIIT) is also considered safe for higher-risk individuals [67], and whilst knowledge gaps remain (including the effects of HIIT on exercise BP directly), HITT has shown efficacy in terms of its BP-lowering potential, with comparable reductions in BP compared to more-moderate-intensity exercise interventions [68,69].

## 6. Re-Measure and Report

### Key Points

- Repeat exercise test with BP measurement;
- Ensure the same exercise testing protocol used pre-intervention is performed, with BP measured at the same fixed submaximal workload at moderate intensity;
- Ensure the same testing equipment is used (including BP monitor) and ideally the same operator performs the test;
- Provide further correspondence surrounding the BP response to patient’s physician.

The exercise professional may conduct a post-intervention exercise test with BP measurement following completion of the exercise intervention. This should be the standard practice to determine effectiveness of the exercise intervention and whether appropriate patient outcomes have been achieved. It is particularly important in the context of the BP management pathway because if the exercise BP was raised pre-intervention, it provides the opportunity to re-assess BP control following the intervention.

When measuring exercise BP during the post-intervention exercise test, it is important to follow the same standardised approach taken pre-exercise intervention. This means using the same exercise testing protocol, equipment, and operator (if possible). Most importantly, BP should again be measured using the same device and at the same fixed workload as the initial exercise test. This is to ensure any change in the BP response is not masked by concomitant improvements in aerobic fitness [21]. It is also recommended that the exercise professional includes detail of the exercise BP response post-intervention in a report to the referring physician.

## 7. The Physician’s Role

The primary care physician plays a critical role in the ongoing management of patients with high BP. Primary care physicians will have oversight of CVD risk management, and so, upon receiving advice of EEBP indicating poor BP control, the primary care physician can follow-up BP management at their discretion. This may include (but is not limited to) physician review of patient home BP measures and/or performing 24-h ambulatory BP measurement, which additionally provides understanding of nocturnal BP control. If the out-of-clinic BP monitoring shows evidence of high BP, initiation of antihypertensive medication (or intensification if the patient is already prescribed medication) may be required. It would be reasonable to expect the primary care physician to provide ongoing correspondence as to the course of action taken (if any) as it relates to BP and CVD risk management with exercise professionals, highlighting anything that may impact ongoing exercise intervention.

## 8. Future Needs

While the clinical pathway recommended in this guiding document has been derived using the best available evidence, there are several evidence gaps that remain in need of clarification. Future research may target the identification of the optimal intensity and workload of exercise BP measurement that elicits EEBP and reveals the presence of high BP through conduct of clinical cohort studies and/or retrospective audits of clinical exercise testing data. Moreover, for the clinical simplicity of this care pathway, a single threshold to denote EEBP at moderate exercise intensity was proffered. However, the optimal EEBP threshold to reveal high BP may be sensitive to certain patient characteristics (e.g., age, sex, cardiorespiratory fitness, and health status), and no one threshold may be appropriate. Optimisation of the intensity of measurement and EEBP threshold would allow more diagnostic accuracy for exercise professionals and primary care physicians in determining a course of action to control high BP. This pathway to identify and control high BP by its nature will likely “do no harm” to patients, exercise professionals, or primary care physicians. However, it remains unknown if it will a lead to changed care pathways, exercise professional-to-primary care physician correspondence, and ultimately improved control of high BP. Certainly future research studies are required to demonstrate its success, which if combined with stakeholder advocacy and societal endorsement should enable practice and educational guideline change.

## 9. Conclusions

High BP is the leading risk factor for CVD, and many cases go unrecognised or remain inadequately controlled. There has been a recent “call-to-action” to influence BP-related patient care amongst allied health and exercise health professions [70], with sufficient clinical evidence suggesting that the measurement of exercise BP can play a central role in identifying BP control. In this paper, we have outlined a simple clinical pathway to achieve this. Exercise professionals conducting exercise testing should measure BP during fixed-workload submaximal exercise (moderate intensity). If exercise systolic BP is raised (≥170 mmHg), uncontrolled high BP should be assumed, prompting correspondence with primary care physicians for follow-up care to ascertain true BP control alongside hypertension-guided exercise and lifestyle interventions to lower CVD risk related to high BP.

## Figures and Tables

**Table 1:** Summary of key recommendations for the best practice measurement of exercise BP.

General:
- Measure BP at a fixed workload during submaximal (moderate) exercise intensity;
- Use manual cuff auscultation or validated automatic BP monitor;
- Ensure correctly fitting cuff size.
For manual measurement:
- Use Korotkoff phase I for systolic BP and phase V for diastolic BP;
- Report BP values within 2 mmHg;
- Avoid excess stethoscope pressure;
- Ensure patient is relaxed through shoulders with additional arm support from operator to hold the arm at heart level;
- Ensure patient does not tightly grip rails (treadmill) or handlebars (bike).

BP, blood pressure. Adapted from Sharman and La Gerche [24].

**Table 2:** Summary of key recommendations for the best practice measurement of home BP.

- Use a validated automatic BP monitor that has been accuracy tested [56]. Instructions on how to check the validation status of a BP device can be found at this website: (https://www.menzies.utas.edu.au/documents/pdfs/Blood-pressure-devices.pdf);
- Use an upper-arm cuff monitor (wrist and wearable devices are not recommended); - Ensure correctly fitting cuff size is applied to a bare upper arm; - Measure BP before eating, taking medications, or vigorous exercise; - Ensure bladder is empty, and refrain from drinking coffee or smoking 30 min prior; - Sit quietly for a minimum of 5 min before taking any readings;Avoid talking while measuring BP; - Sit with feet flat on the floor, back and arm supported in a relaxed position; - Measure BP each day for 7 days (minimum 5); - Perform readings twice daily: in the morning and in the evening; - Perform 2 measurements each time, with 1 minute between measures; - Record reading in a diary and bring to next physician appointment

BP, blood pressure. Adapted from Parati et al. [54] and Sharman et al. [55].

## Data Availability

Not applicable.

## Supplementary Materials

The following supporting information can be downloaded at: https://www.mdpi.com/article/10.3390/ijerph19052819/s1, Figure S1: Pathway to identify and control high blood pressure in clinical exercise settings; Figure S2: Example correspondence from an exercise professional to a primary care physician.
