# Supplementary material for: The Identification and Management of High Blood Pressure Using Exercise Blood Pressure: Current Evidence and Practical Guidance
Source: Int J Environ Res Public Health. 2022 Feb 28;19(5):2819. doi: 10.3390/ijerph19052819 (PMC8910717; doi:10.3390/ijerph19052819)
Supplement: Supplementary file 1 [file ijerph-19-02819-s001.zip › ijerph-1512709-supplementary.pdf]

Pathway to identify and control high blood pressure in clinical exercise settings

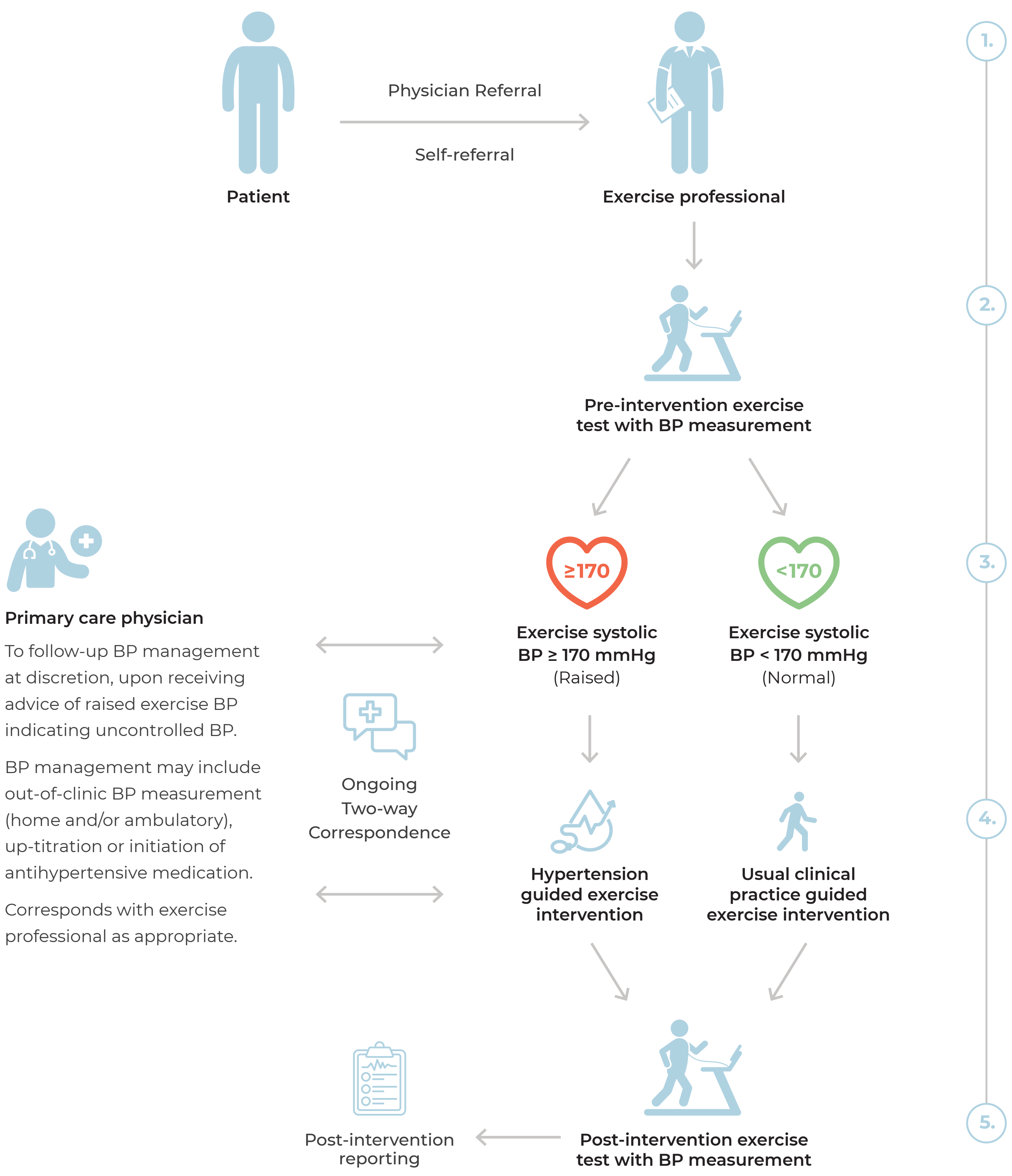

1. **Initial consultation**

- Establish health status and appropriateness of exercise/physical activity. intervention
- Determine if patient is to undergo aerobic exercise test.
- Ensure sufficient consultation time to allow thorough assessments.

2. **Measure**

- Perform an aerobic exercise test with consideration of contraindications.[1]
- Exercise test may follow a maximal or submaximal protocol of any modality.
- Measure BP according to recommendations,[2] at a ***fixed submaximal workload*** of moderate intensity.

3. **Evaluate and correspond**

- If exercise systolic BP is raised, assume uncontrolled BP and:
  a. Provide detailed correspondence to physician
  b. Encourage patient to discuss BP with a physician
  c. Encourage patient to perform home BP monitoring according to guidelines[3]
  d. Proceed with exercise intervention.

4. **Intervene**

- If exaggerated exercise BP is evident (assume uncontrolled high BP), the exercise intervention should be guided by current recommendations for exercise and hypertension.[4]
- If exercise systolic BP is normal, the exercise intervention should be guided by usual and appropriate clinical practice.

5. **Re-measure and report**

- Repeat exercise test with BP measurement
- Ensure to the same exercise testing protocol used pre-intervention is performed, with BP measured at the same fixed submaximal workload of moderate intensity
- Ensure the same testing equipment is used (including BP monitor) and ideally the same operator performs the test.
- Provide further correspondence surrounding the BP response to patient's physician.

[1]American College of Sports Medicine: Guidelines for exercise testing and prescription, 10th ed: Lippincott, Williams & Wilkins.
[2]Sharman JE and LaGerche A. J Hum Hypertens. 2015;29:351-8.
[3]Parati et. al., J Hypertens. 2021; 1;39(9):1742-1767.
[4]Exerciseismedicine.com.au/wp-content/uploads/2021/04/EIM-FactSheet_Hypertension_2020.pdf

Figure S1: Pathway to identify and control high blood pressure in clinical exercise settings.

(Date)

Dear *(Dr name),*

RE: *(full patient name), (date of birth)*

Thank you for referring *(name)* for exercise and lifestyle intervention related to *(health condition).*

On *(date), (name)* completed a submaximal exercise test as part of an initial assessment undertaken prior to commencing an exercise training intervention.

Blood pressure (BP) was recorded at rest (following guidelines) and in a standardised manner during a moderate intensity of exercise (values are reported in table below).

	**Recorded values**	**Reference range***	**Interpretation**
**Resting BP**	136/85 mmHg	<140/90 mmHg	Within normal limits
**Exercise BP**	189/90 mmHg	Systolic BP <170 mmHg	Above normal limits

Recent research evidence suggests exaggerated (elevated) exercise BP is associated with increased risk of high BP-related cardiovascular disease that may not have been picked up from normal measures of BP in the clinic at rest.[1]

Although *(name's)* BP can be considered within normal limits at rest, the value of BP during exercise indicates a potential lack of BP control that may warrant further investigation via out-of-clinic BP measures.

I have therefore encouraged *(name)* to complete home BP monitoring according to guidelines,[2] and they will bring the completed diary to their next appointment with you for review.

I will be proceeding with an exercise intervention that assumes the presence of uncontrolled high BP, and as such would also appreciate any correspondence from you about the course of action you have taken (if any) to monitor or manage *(names)* BP.

Kind regards,

(Exercise professional name)

1.Schultz MG, La Gerche A and Sharman JE. Blood Pressure Response to Exercise and Cardiovascular Disease. Current hypertension reports. 2017;19:89.

2.Sharman JE, Howes FS, Head GA, McGrath BP, Stowasser M, Schlaich M, Glasziou P and Nelson MR. Home blood pressure monitoring: Australian Expert Consensus Statement. J Hypertens. 2015;33:1721-8.

** Note the reference range for classifying high blood pressure differs in the United States of America, with high blood pressure considered as ≥130/80 mmHg.*

Figure S2: Example correspondence from an exercise professional to a primary care physician.
